# Nursing as a Collegiate Course

**Published:** 1908-07-18

**Authors:** 


### Nursing Administration.

### NURSING AS A COLLEGIATE COURSE.

There is undoubtedly a tendency among enthusiastc superintendents of nurses to desire an enlargement of the nurse's curriculum. It seems to them that the things commonly deemed necessary for the equipment of a trained nurse are too few and simple, and that intellectually there is scope in her training for more study. They would extend the nurse's sphere in various directions, and make the profession one which would develop the mental as well as the moral and technical faculties of the women who engage in it. In America this feeling has found expression in the founding of a collegiate course at the University of Columbia, so that women desirous of taking a degree may graduate in nursing. The result so far has not been altogether encouraging to the promoters of the scheme, for the expense appears to be prohibitive, and but few women have hitherto availed themselves of the advantages offered. The immediate outcome of the movement in favour of stimulating the nurse's mental preparation for her duties has been to add to the lectures of probationers to a deplorable extent and cumber their memories with a mass of ill-digested medical details which can serve little purpose in fitting them for their special duties in the sick-room. In England the conservative methods of the old-established hospitals have restricted experiment with the nurse's curriculum within narrow limits. But while attention is rightly concentrated in this country far more on making instruction thorough than on increasing the number of subjects taught, some are still left wondering to what extent it is legitimate in the interests of nursing in general to endeavour to raise the standard of required knowledge and induce women to enter upon the study of nursing with some of the intellectual ardour with which they would approach the study of music, political economy, or medicine itself. It is the peculiarity of nursing that it stands as handmaiden to other professions, and cannot develop far in any direction without trenching on some other domain. The nurse needs a clear grasp of elementary physiology, but even a knowledge of the subject such as is required from a first-year student of medicine is of no conceivable use to her. She is constantly handling drugs and must be acquainted with the names and properties of those most commonly in use, but she has no need for any systematic study of the materia medica. And it is the same with every other branch of knowledge within her range. She must know a little about cooking and diets, but she need not be able to turn out entrées nor to lecture on dietetics. She ought to understand the principal conditions tending to recovery in her patient's environment, but she need not study hospital architecture. She should, if she undertakes district nursing, have some knowledge of the principal measures relating to public health, but she need not study advanced sociology nor plunge into the mazes of the factory laws. In whatever direction she looks she is called back from abstract study to the realisation of her patient's needs, and must never stray very far from practice into the realms of theory and research. We believe this to be a source of strength, not a source of weakness, to nurses. It is their vocation to carry out and apply for the alleviation of suffering humanity the discoveries which men and women who have chosen other branches of work succeed in placing at their disposal. In the pursuance of their duties nurses need all the science, all the efforts and researches which the twentieth century can bring. It is their part to take from all for the patients' necessities, to learn from all that their task may be well performed, loyally to obey those who have studied to effect cure, and thus to use the brains of other people to advance their own branch of work. But they cannot do two things at one time. They cannot be leaving their patients to study the causes of their disease. They cannot desert their district to sit on committees and investigate the causes of poverty. They cannot leave the sick man hungry while they make chemical experiments relating to diet. They have chosen their part, and it is the part of personal service, one worthy of their best energies and admitting in duty hours of no rival. Hence we must conclude that those for whom book work has overwhelming attractions, who weary of the sick-bed, and only wake to keenness in the lecture-room, have mistaken their vocation. It will not exalt the profession of nursing to make it an academic subject. It will merely induce the erroneous impression that nursing need not absorb the whole energies of the women who undertake it, and encourage an amateur dabbling in the scientific pursuits which border nursing, but lie outside its own special province. Every attempt to divorce nursing from the patient and render it a field for the display of ambition is a tacit slur on the dignity of personal service as shown round the bedside of the sick. To encourage proficiency in the subjects necessary to be mastered by the probationer, and within the comprehension of average abilities, by the bestowal of prizes and [illegible]edals is now the wise policy of all the best traini[illegible] schools. It is a very different thing to strive after [illegible] standard of attainments only to be reached by [illegible]omen of leisure, means, and exceptional facility for book work. Not in that direction does the perfecting of the nursing curriculum lie. No one is altogether satisfied with the nurse's preparation for her future work as carried out in the majority of hospitals in the present day, but enough has already been done to show that nursing demands the concentration of every faculty on its own wide domain, and must lose rather than gain in distinction by trespass on the highly specialised branches of knowledge surrounding it. There is room for development and for patient experiment. There is still much to be done before the nurse steps from her training school equipped at all points for the duties which lie before her, with a solid groundwork, in place of a mere smattering of her subjects.

